# Analysis of AML genes in dysregulated molecular networks

**DOI:** 10.1186/1471-2105-10-S9-S2

**Published:** 2009-09-17

**Authors:** Eunjung Lee, Hyunchul Jung, Predrag Radivojac, Jong-Won Kim, Doheon Lee

**Affiliations:** 1 Department of Bio and Brain Engineering, KAIST, Daejeon 305-701, South Korea; 2 Biomedical Research Center, KAIST, Daejeon 305-701, South Korea; 3 School of Informatics, Indiana University, Bloomington, IN 47408, USA; 4 Department of Laboratory Medicine and Genetics, Samsung Medical Center, Sungkyunkwan University, School of Medicine, Seoul 135-710, South Korea

## Abstract

**Background:**

Identifying disease causing genes and understanding their molecular mechanisms are essential to developing effective therapeutics. Thus, several computational methods have been proposed to prioritize candidate disease genes by integrating different data types, including sequence information, biomedical literature, and pathway information. Recently, molecular interaction networks have been incorporated to predict disease genes, but most of those methods do not utilize invaluable disease-specific information available in mRNA expression profiles of patient samples.

**Results:**

Through the integration of protein-protein interaction networks and gene expression profiles of acute myeloid leukemia (AML) patients, we identified subnetworks of interacting proteins dysregulated in AML and characterized known mutation genes causally implicated to AML embedded in the subnetworks. The analysis shows that the set of extracted subnetworks is a reservoir rich in AML genes reflecting key leukemogenic processes such as myeloid differentiation.

**Conclusion:**

We showed that the integrative approach both utilizing gene expression profiles and molecular networks could identify AML causing genes most of which were not detectable with gene expression analysis alone due to the minor changes in mRNA level.

## Background

Mining disease-causing genes and elucidating their pathogenic molecular mechanisms are of great importance for developing effective diagnostics and therapeutics [1-5]. Along with many genetic and genomic studies aimed at identification of disease genes (e.g. linkage analysis, cytogenetic studies, microarray experiments, proteomic studies), several computational methods have been proposed to prioritize candidate genes based on various information including sequence similarity, literature annotation, and molecular pathways [6-11]. Given a set of genes known to be involved in disease, these methods typically score similarities between candidate genes and known disease genes in terms of various genomic features.

Recently, accumulated knowledge about molecular interaction networks in human cells such as protein-protein, and protein-DNA interactions has been utilized to predict disease genes [6-8,10,12-14]. The previous studies have incorporated topological characteristics of known disease genes such as degrees in networks [14], the overlap between interaction partners of candidate genes and those of known disease genes [6], the probability of candidate genes to participate in the same protein complexes with known disease-causing genes [10], or the distribution of distances from candidate genes to known disease genes [13].

Despite their successful performance in general, for some specific diseases of our interest, such as acute myeloid leukemia (AML), the performance is not satisfactory (AUC = 0.55 by Radivojac et al. [13]). We hypothesized that integrating molecular networks with mRNA expression profiles from patients might help delineate disease-specifically dysregulated molecular subnetworks containing disease-causing mutation genes. Chuang et al. supported this hypothesis showing the identified subnetworks included significantly enriched known breast cancer mutation genes [15]. Mani et al. proposed another method predicting oncogenes in B-cell lymphomas integrating both molecular interactions and mRNA expressions [16].

Here, we identified molecular subnetworks dysregulated in AML patients which were associated with key leukemogenic processes such as myeloid differentiation. We also evaluated the enrichment of known AML-causing mutation genes within the subnetworks, and found that the subnetworks contain significant fraction of known AML genes (mostly non-differentially expressed) embedded among the interconnections of differentially expressed genes. In addition, several characteristics of AML genes in the subnetworks were reported in this study, which can be utilized to build prediction models for unknown AML genes.

## Results and discussion

### Identification of subnetworks perturbed in AML

**Figure 1:**
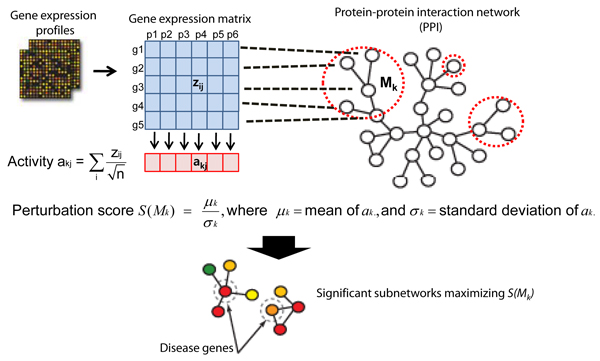
**Schematic overview of the subnetwork identification**. Schematic overview of the subnetwork identification. The mRNA expression levels of each gene were overlaid on its corresponding protein in the network and subnetworks whose combined activities across the patients have high perturbation score were searched. An activity level (*a*_*kj*_) for a subnetwork *M*_*k *_in *j*^th ^sample was defined as the mean expression levels with the square-root of the number of participating genes in the denominator. The perturbation score *S*(*M*_*k*_) for the subnetwork was then calculated as the mean over the standard deviation of the activity levels across patients.

The method to find subnetworks of AML is similar to that of our previous work [15], and visualized in Figure 1. We overlaid the gene expression values of each gene on its corresponding protein in the protein-protein and protein-DNA interaction network and searched for subnetworks whose combined activities across the patients have high perturbation scores (PS) starting from each node in a greedy fashion. The gene expression profiles used cDNA platforms where each expression value of gene *g*_*i *_in patient *p*_*j *_(*g*_*ij*_) is the mean log ratio of intensities from Cy5-labeled mRNA of the patient and Cy3-labeled reference mRNA. Expression values were normalized for each gene across patients to have mean 0 and standard deviation 1 (*z*_*ij*_). We took absolute values of expression levels to measure perturbation effect regardless of the direction of changes (i.e. up or down). The perturbation score was defined as the mean over standard deviation of an activity vector across samples where each activity value was the averaged expression level of genes participating in each subnetwork *M*_*k *_and is denoted as *S(M*_*k*_) in Figure 1. Subnetworks with higher mean and smaller variance of activity levels are considered more perturbed in AML samples.

### AML subnetworks associated with key leukemogenic processes

**Figure 2:**
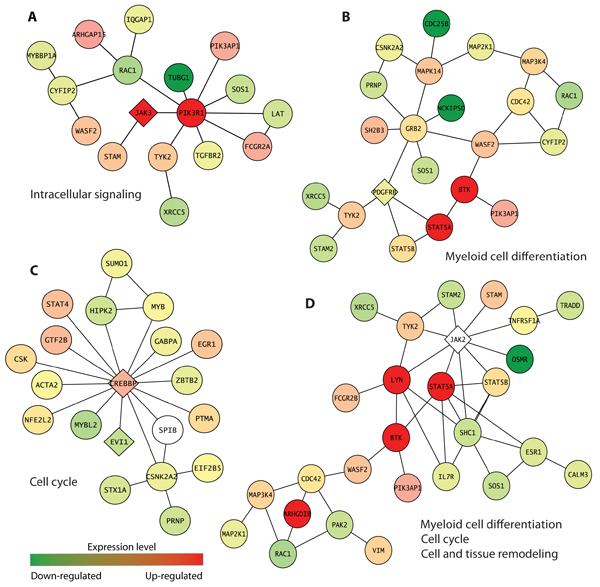
**Examples of subnetworks containing known AML mutation genes**. Nodes and links represent human proteins and protein interactions, respectively. The color of each node shows the degree of mRNA level change in AML patients. Known AML mutation genes are marked with the diamond shape.

Through the search for sutnebworks perturbed in AML patients, we identified 269 subnetworks (p < 0.05) comprising of 859 genes whose functions are associated with AML development processes such as myeloid differentiation, cell signaling of growth and survival, cell cycle, cell and tissue remodeling. Within the significant AML subnetworks, we found many of already known AML-causing mutation genes. Figure 2 shows examples of subnetworks containing known AML genes such as JAK2, JAK3, PDGFRB, and CREBBP, and their representative biological processes. Especially, a severe block in myeloid differentiation is known to be the hallmark of AML.

### AML subnetworks enriched for known AML causing genes

**Figure 3:**
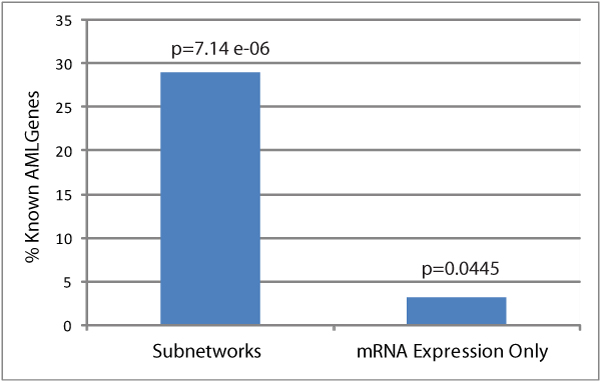
**The enrichment of AML mutation genes in subnetworks**. 18 out of 62 AML genes (29.03%) were found in 269 subnetworks including 859 genes, and their enrichment was significant (p-value P = 7.14e-6) through the hypergeometric test (the probability of 18 AML genes out of all 62 are found in the subnetworks including total 859 genes out of 9142 genes in the whole network). In contrast, only two AML genes (FLT3, JAK3) (3.23%) were found among 859 top differentially expressed genes in their mRNA levels (P = 0.04)

We have evaluated the enrichment of known AML genes in significant subnetworks in a systematic way. We compiled 62 genes known to be causally mutated in AML from Sanger Cancer Gene Census. 150 out of 269 subnetworks included at least one AML gene, and 49 subnetworks included two or more AML genes. As shown in Figure 3, subnetworks were much more significantly enriched for AML causing mutation genes than the conventional gene-expression analysis alone without considering molecular interactions (p value P = 7.14e-6 vs. P = 0.04).

### Characteristics of AML genes in the subnetworks

Table 1 lists 18 known AML genes detected in perturbed subnetworks along with the number of subnetworks including a designated gene and the magnitude of differential expression in its mRNA level (DES) for each gene. JAK3, KIT, EVI1, and CREBBP appeared in more than 10 subnetworks while other genes were present in two or mostly one subnetwork. JAK3 with the extremely high frequency (110) has been reported to have great biological importance in AML pathogenesis through gain-of-function JAK3 mutations (e.g. JAK3A572V, JAK3V722I, JAK3P132T) activating signal transduction [17]. Mutations in KIT having the second highest frequency (43) were also found in more than 30% of patients with de novo AML [18]. The appearance frequency of an AML gene in subnetworks was more correlated with the magnitude of its DES score (correlation coefficient *r *= 0.43) than the number of interacting partners, the node degree (*r *= 0.01).

**Table 1:** AML mutation genes in subnetworks

Genes	Number of Subnetworks	Degree	DES^+^
JAK3*	110	32	2.73
KIT	43	54	1.97
EVI1	16	7	-1.27
CREBBP	14	209	1.5
EP300	7	216	2.36
BCR	2	32	1.41
FLT3**	2	11	3.58
NSD1	2	6	1.14
PTPN11	1	109	-1.07
JAK2	1	87	N/A
PDGFRB	1	56	-0.33
NPM1	1	33	-0.37
RUNX1	1	22	0.68
GATA2	1	20	1.58
PICALM	1	8	0.44
FNBP1	1	7	2.05
RPL22	1	3	1.08
TRIP11	1	2	-1.29

^+^DES means the degree of change in its mRNA level for each gene.

Genes with the absoluteDES ranked within top 5% are marked with **, and genes within top 10%  are marked with *.

**Figure 4:**
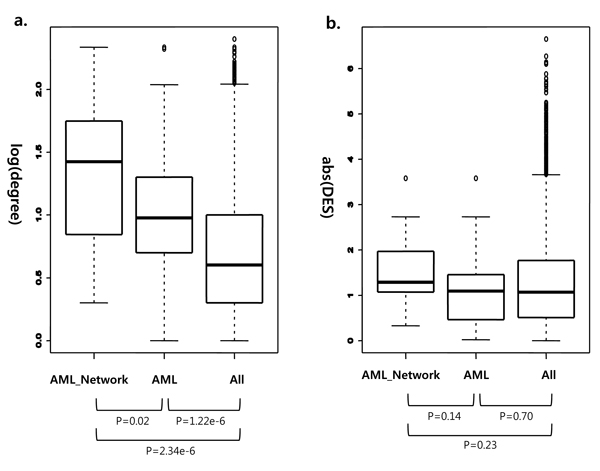
**(a) Degrees and (b) mRNA expression changes of AML genes**. Each figure shows node degrees and magnitudes of differential expression (DES) for AML-causing mutation genes found in subnetworks (AML_Network), all known AML mutation genes (AML), and all genes in the whole network. The bottom and top of each box are first and third quartiles, and the band near the middle of the box is the median. Whiskers extend to at most 1.5 times the inter-quartile range. Beyond the whiskers, all outliers are shown in open circles. The statistical significances for differences between two groups of genes (e.g. AML_Network vs. All genes) measured by non-parametric Wilcoxon rank-sum test are denoted below the labels of gene groups.

We examined whether AML genes captured in subnetworks might have high degrees in the network because that property has been used to predict unknown disease genes in other diseases previously (Figure 4a). The figure shows that all known AML-causing genes (AML) and AML genes captured in subnetworks (AML_Network) have significantly more interaction partners than all genes in the network (P = 1.22e-6, and P = 2.34e-6, respectively). AML genes found in subnetworks have slightly higher degree than AML genes not captured in subnetworks (P = 0.02). The mean and median degrees of all genes in the network are 9 and 4, while those of 18 AML genes are 51 and 27. Though this result supports that known AML genes have tendency of high network degrees, low degree AML genes such as RPL22, and TRIP11 also appeared in the subnetworks.

Finally, we investigated the differential expression of AML genes in mRNA levels (Figure 4b). There was no significant difference between each group of genes, and all known AML genes and those found in subnetworks except FLT3, and JAK3 did not show mRNA level aberrations. This result shows that gene expression alone does not provide enough information to predict unknown AML-causing mutation genes. However, our integrative approach could capture non-differentially expressed AML genes in subnetworks if they were entangled with differentially expressed neighbour proteins yielding subnetworks with high perturbation scores.

## Conclusion

We have demonstrated that integration of condition-independent molecular networks extracted from various types of cells and experiments under different conditions, and disease-specific mRNA expression profiles of AML patients enables the dissection of pathogenic modules of interacting proteins reflecting key leukemogenic processes. In addition, the dissected modules are enriched for AML-causing mutation genes most of which are not detectable with gene expression analysis alone due to minor changes in their mRNA levels. Identification of subnetworks perturbed in AML patients can provide novel molecular hypotheses underlying AML etiology, and investigated characteristics of known AML genes appearing in the subnetworks can be exploited to predict unknown AML-causing genes.

## Methods

### Protein-protein interaction networks

We downloaded the PPI network from the PhenoPred website by Radivojac et al. [19]. It consists of 41456 physical interactions among 9142 proteins assembled from Human Protein Reference Database (HPRD) [20], the Online Predicted Human Interaction Database (OPHID) [21], and studies by Rual et al. and Stelzl et al. [22,23].

### mRNA expression profiles of AML patients

Gene expression profiles of 65 peripheral-blood samples and 54 bone marrow specimens from 116 adult patients with AML were downloaded from Gene Expression Omnibus (GSE425) whose expression values are log ratios (base 2) of mean intensities of patient samples vs. common reference mRNA [24]. Gene identifiers of three cDNA microarray platforms (GPL317,318,319) were mapped to gene symbols using accompanied gene annotation files from GEO yielding 6987 gene symbols with expression levels in at least one of three platforms.

### Mutation genes in AML patients

We compiled two sets of AML-associated genes: 14 genes downloaded from PhenoPred web site originally collected from OMIM [25], Swiss-Prot [26], and HPRD [20] by Radivojac et al. (Disease Ontology ID: 9119) [19], and 62 genes whose somatic and germline mutations are causally implicated in AML patients downloaded from Sanger Cancer Gene Census [27], and also appearing in our PPI network.

### Significance evaluation of subnetworks

To evaluate the significance of the identified subnetworks, we performed the same search procedure over 1000 random trials in which the expression vectors of individual genes are randomly permuted in the network. The p value of each real subnetwork was calculated as the fraction of random subnetworks having higher PS scores than the designated real subnetwork among all random subnetworks. We considered subnetworks with the p-value P < 0.05 significant in this work.

## Competing interests

The authors declare that they have no competing interests.

## Authors' contributions

EL designed the study, and analyzed the results. EL and HC carried out the experiments. EL wrote the initial draft of the manuscript. HC, PR, JK, and DL revised the manuscript, and gave valuable suggestions and improvements. DL supervised this work. All authors read and approved the final manuscript.
